# Bone Marrow Involvement in Melanoma. Potentials for Detection of Disseminated Tumor Cells and Characterization of Their Subsets by Flow Cytometry

**DOI:** 10.3390/cells8060627

**Published:** 2019-06-21

**Authors:** Olga Chernysheva, Irina Markina, Lev Demidov, Natalia Kupryshina, Svetlana Chulkova, Alexandra Palladina, Alina Antipova, Nikolai Tupitsyn

**Affiliations:** FSBI “N.N. Blokhin Russian Cancer Research Center“ of Ministry of Health of the Russian Federation, 115478 Moscow, Russia; irina160771@yandex.ru (I.M.); nntca@yahoo.com (L.D.); natalya-2511@yandex.ru (N.K.); chulkova@mail.ru (S.C.); alexandra.93@mail.ru (A.P.); a.s.antipova@gmail.com (A.A.); nntca@yahoo.com (N.T.)

**Keywords:** bone marrow, melanoma, disseminated tumor cells, solid cancers, single-cell analysis, enrichment and detection technologies, flow cytometry, tumor stem cells, HMB-45, CD133

## Abstract

Disseminated tumor cells (DTCs) are studied as a prognostic factor in many non-hematopoietic tumors. Melanoma is one of the most aggressive tumors. Forty percent of melanoma patients develop distant metastases at five or more years after curative surgery, and frequent manifestations of melanoma without an identified primary lesion may reflect the tendency of melanoma cells to spread from indolent sites such as bone marrow (BM). The purpose of this work was to evaluate the possibility of detecting melanoma DTCs in BM based on the expression of a cytoplasmatic premelanocytic glycoprotein HMB-45 using flow cytometry, to estimate the influence of DTCs’ persistence in BM on hematopoiesis, to identify the frequency of BM involvement in patients with melanoma, and to analyze DTC subset composition in melanoma. DTCs are found in 57.4% of skin melanoma cases and in as many as 28.6% of stage I cases, which confirms the aggressive course even of localized disease. Significant differences in the groups with the presence of disseminated tumor cells (DTCs^+^) and the lack thereof (DTC^−^) are noted for blast cells, the total content of granulocyte cells, and oxyphilic normoblasts of erythroid raw cells.

## 1. Introduction

Today oncology is a rapidly developing field of medicine. Every year novel target and immunological agents acting against cancer at the molecular level are added to clinical oncologists’ practice, and many such agents are currently at various stages of clinical development. However, notwithstanding significant progress over the last decade and a broad variety of therapeutical options, several fundamental questions remain to be answered: What are causes of cancer development? What are mechanisms of metastasis and recurrence? At what stage of disease development can we influence these processes?

Over the last 150 years there were many theories to explain processes developing both in the tumor and in the patient’s body. By the end of the first quarter of the 21st century the world medical community has passed a long way from the first publication by T.R. Ashworth in the *Medical Journal of Australia* in 1869 [1], where the author described for the first time circulating tumor cells in a cancer patient, and the ‘seed and soil’ theory proposed by Stephen Paget in 1889 [2], through the theory of late dissemination (linear progression) by William Stewart Halsted [3,4,5] to the theory of early metastasis (parallel progression) by Christophe Klein [6] and the concept of the premetastatic niche by Bethan Psaila and David Lyden in 2009 [7]. The key question in all of these theories was how tumor cells managed to overcome immune surveillance [8], to preserve their proliferative potential and to proliferate in alien environments even after several decades of latency [9,10].

It seems natural that bone marrow (BM) with its advanced capillary network and a cocktail of soluble protein factors, integrins, chemokines, cell adhesion molecules, and a variety of growth factors is the most attractive niche for tumor cells [11,12]. Being basically alien, BM makes its environment appropriate for disseminated tumor cell (DTC) persistence via sophisticated antigenic, immunogenic, and cellular mechanisms [13,14]. DTCs may have different fates in a new microenvironment. Most of them die within several weeks or months [15], while DTCs preserving their vitality without decrease or increase in their total number may enter latency and form so called dormant metastases.

Dormant tumor cells have three main differences from other tumor cells, i.e., the ability to survive in alien and even hostile environments for a long time, temporary but reversible growth arrest, and resistance to target cytostatic agents [16]. These DTC properties make them biologically closer to tumor stem cells, a minor primary tumor subset seeming to play a leading role in the self-maintenance and metastasis of malignancies [17].

BM involvement is described in multiple non-hemopoietic neoplasms and is shown to be an independent poor prognostic factor for overall and disease-free survival [18,19,20,21,22,23]. Interestingly, these publications mainly address cancers of the breast, stomach, lung, colon, or prostate, while studies of melanoma are few and require further analysis.

Observations of hematogenous metastases from melanoma after 10 [24] or even 40 [25] years after removal of the primary tumor and frequent melanoma manifestations without an identified primary may reflect melanoma cell tendency to spread from indolent sites [26,27] such as BM.

gp100—HMB-45, a cytoplasmatic premelanocytic glycoprotein is a reliable marker of melanoma cells. It was discovered as one of the first melanoma antigens to demonstrate high sensitivity (up to 93%) and specificity (up to 100%) [28] and is usable to identify DTCs.

The Hemopoiesis Immunology Laboratory (N.N. Blokhin Cancer Research Center, Russian Federation Health Ministry) has developed a procedure to identify DTCs by flow cytometry [29]. Flow cytometry has certain advantages as compared to cytology, immunohistochemistry, and molecular biology techniques. For instance, contemporary multicolor flow cytometry can analyze 12 or more parameters in a single cell and accumulate a large number of events with sensitivity close to that of PCR (10^–4^ to 10^–6^) and allows most complete description of the DTC immunophenotype [30]. Besides pure quantification of DTCs, flow cytometry therefore helps to study DTC subsets such as tumor stem cells or to identify surface molecular targets for drugs (Her2/neu, PDL1).

The purpose of this work was to evaluate the possibility of detecting melanoma DTCs in BM based on the expression of HMB-45 using flow cytometry, to determine the frequency of BM involvement in patients with melanoma, to analyze DTC subset composition in melanoma as to the expression of CD56 and CD57 that were an additional criterion for melanoma immunological diagnosis, and to assess the proportion of tumor stem cells among DTCs based on the presence of CD133.

## 2. Materials and Methods

A total of 47 patients (23 males and 24 females) aged 20–72 (median 49.8) years managed at the N. N. Blokhin Russian Cancer Research Center for skin melanoma during 2018–2019 were enrolled in the study. The diagnosis was verified histologically in all patients. This study was approved by the institutional ethical committees (Local ethical committee N. N. Blokhin Russian Cancer Research Center of Ministry of Health of the Russian Federation; UDC 616-006, Reg. № AAAA-A16-116122210071-4, Inv. 479.) and was done with the informed consent of the patients. Most of the patients (42.6%) had stage IV disease based on complex examination. BM involvement was assessed by morphology and immunology at diagnosis. Table 1 demonstrates patient distribution by stage.

Morphological examination included myelogram count and identification of tumor cells on six Romanovsky-stained bone marrow smears by two morphologists in parallel. Immunological identification of DTCs in BM was done by flow cytometry. Samples were lysed using BD FACS lysing solution (Beckton Dickinson, Franklin Lakes, NJ, USA), then washed in phosphate-buffered saline (PBS), and re-suspended in 100 mL of PBS. Cells were incubated for 15–20 min in the dark at room temperature together with a cocktail of monoclonal antibodies directly conjugated with fluorescein isothiocyanate (FITC), phycoerythrin (PE), allophycocyanin (APC), and Horizon V500 and Horizon V450 fluorochromes (Table 2). All samples were processed within 24 h after collection. Antibody labeling was measured by multiparameter flow cytometry using FACS Canto II (Beckton Dickinson). Twenty million myelokaryocytes (or all cells in the sample) were collected to identify DTCs. Tumor cells were detected by the lack of expression of the common leukocyte antigen CD45 in combination with bright expression of HMB-45. To identify DTC subpopulations expression of CD133, CD56, and CD57 molecules was analyzed among the CD45^–^HMB-45^+^ cells.

Results were analyzed using Kaluza Analysis v2.1 (Beckman Coulter, Brea, CA, USA) software. Statistical analysis of data used IBM-SPSS Statistics v.17 package (IBM, Armonk, NY, USA).

## 3. Results

Morphological analysis of BM biopsies included myelogram count and tumor cell identification.

In the analysis of hematopoiesis, we excluded cases with bone marrow dilution with peripheral blood. Comparison of the average bone marrow parameters according to the myelogram is shown in the Table 3.

Significant differences in the groups with the presence of disseminated tumor cells (DTCs+) and the lack thereof (DTC−) were noted for blast cells, the total content of granulocyte cells, and erythroid germ indicators.

The level of blast cells was higher in patients with no DTCs: 1.46% ± 0.14% (*n* = 20) and 1.1% ± 0.09% (*n* = 25), *p* = 0.026.

On the contrary, the total content of granulocyte cells was higher in patients with DTCs in the BM: 65.4% ± 1.4% (*n* = 25) and 60.8% ± 1.5%: (*n* = 20), *p* = 0.025.

The most significant differences were obtained with respect to cells of the erythroid series. Thus, the percentage of oxyphilic normoblasts was significantly higher in patients with no DTCs in the BM: 9.1% ± 0.88% (*n* = 20) and 6.3% ± 0.52% (*n* = 25), *p* = 0.006. It should be noted that, in the group as a whole, the levels of oxyphilic normoblasts were increased compared to the norm in 67% of patients. Accordingly, the sum of nucleated erythroid cells was also higher in melanoma patients with no DTCs in the BM: 21.5% ± 1.4% (*n* = 20) and 17.4% ± 1.3% (*n* = 25), *p* = 0.042. This was reflected in a significantly higher leuco–erythroid ratio in patients with the presence of DTCs in the bone marrow: 5.6% ± 0.6% (*n* = 25) and 4.0% ± 0.4 (*n* = 20), *р* = 0.034.

It is interesting to note that when analyzing according to the tables of conjugacy of characters, only two indicators of the myelogram were reliably associated with the presence of DTCs in the BM—the total content of granulocyte cells and the level of oxyphilic normoblasts.

The relationship of DTCs with the total amount of granulocyte cells consisted in the fact that, in the presence of DTCs, a decrease in the total level of granulocyte cells was observed in only 8% of cases, while in the absence of DTCs a decrease in granulocyte cells was observed in 30% of cases, chi-square = 8.9; *p* = 0.012.

**Figure 1:**
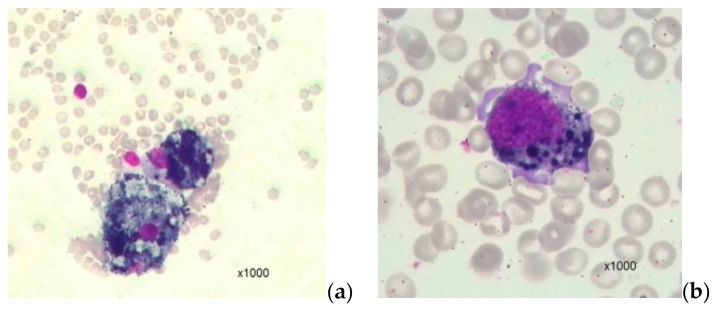
Melanoma disseminated tumor cells in bone marrow. This figure presents a case of detection of skin melanoma cells in bone marrow punctate ((**a**) and (**b**), ×1000 magnification). Punctate bone marrow is poor. Normal lines of myelopoiesis are depressed. Cell complexes of a non-hemopoietic nature are determined. Additionally, there are scattered, separately lying tumor cells. They are represented by cells of a large size, and basophilic colored pigment granules of various sizes are visible in the cytoplasm. The morphological picture of the bone marrow is characteristic of metastatic lesions in melanoma.

A different situation was noted with respect to oxyphilic normoblasts, whose normal content in the absence of DTCs was observed in 15% of cases, and in the presence of DTCs—three times more often—in 44% of cases. On the contrary, an increase in the level of oxyphilic normoblasts in the absence of DTCs occurred in 85% of cases, in the presence of DTCs, in 56%, chi-square = 4.4; *p* = 0.037. Melanoma cells were identified in BM by morphology in one of 47 cases only (Figure 1).

Immunological analysis of DTCs in BM was based on a threshold of one tumor cell (Syto41^+^CD45^−^HMB-45^+^) per 10 million myelokaryocytes. A mean of 14,146,987 (±957,728) myelokaryocytes were analyzed in each sample. DTCs were found in 57.4% of BM samples (*n* = 27) based on the threshold level. Interestingly, flow cytometry of melanoma cells has specific features due to morphological characteristics of these cells such as a rather large size and the presence of pigmented inclusions of various diameters (from dust-like to large fused granules of different diameter). For instance, the melanoma DTCs have high direct and side light scatter characteristics and require adequate protocols for flow cytometer tuning.

There were no significant differences in DTC counts with respect to gender, age, or disease stage. What is important is that BM involvement was discovered at all disease stages (Table 4). This means that hematogenic tumor cell dissemination occurs already from clinically localized disease.

**Figure 2:**
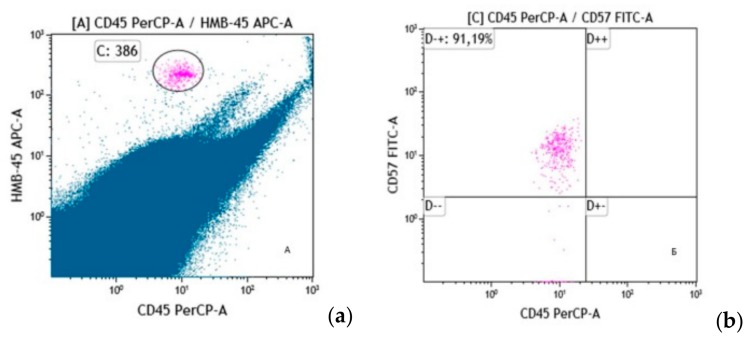
Disseminated tumor cells of skin melanoma as identified by immunological flow cytometry. This figure shows an example of detection of skin melanoma DTCs. On the cytogram (**a**) in gate C, DTCs were observed on the basis of the bright expression of HMB-45 (y-axis) and the absence of CD45 expression (x-axis). On the cytogram (**b**), the analysis of the subpopulation composition of DTCs in melanoma was performed in relation to the expression of the CD57 antigen. Cells are characterized by distinct CD57 expression (y-axis) and lack of CD45 expression (x-axis). Most DTCs (91.19%) are CD57+.

DTCs were additionally characterized by CD56 and CD57 expression. In our study, CD56 and CD57 expression was assessed in 23 BM samples. Among them, DTCs were present in 54.2% (*n* = 13) though these cells did not express CD56. CD57 expression on DTCs was found in six cases (46.2%) (Figure 2). Importantly, not all 100% of DTCs in each BM sample demonstrated CD57 expression. On average 87.4% ± 5.8% of DTCs were CD57-positive. Of interest, 50% of CD57+ patients had stage IV, two of six had stage III, and one patient had stage IIc disease.

A minor tumor stem cell (TSC) subset with maximum resistance to conventional anticancer therapies plays a special role in metastasis. According to the literature, melanoma TSCs are characterized by expression of antigens such as CD44, CD271, and CD133. We identified TSCs among melanoma DTCs by CD133 expression.

**Figure 3:**
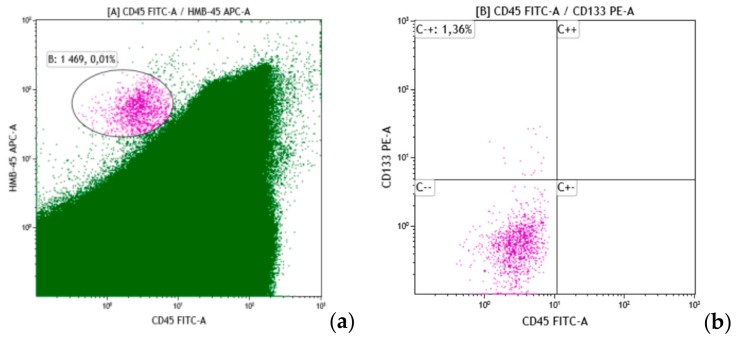
Identification of CD133-positive DTCs. This figure shows an example of the assessment of DTC subpopulations by the tumor stem cell marker CD133. On the cytogram (**a**), 0.01% DTCs was detected by the distinct expression of HMB-45 (*y*-axis) and the lack of expression of the pan-leukocyte antigen CD45 (x-axis). Within the DTCs, expression of CD133 evaluated. Cytogram (**b**) (*x*-axis is CD45, *y*-axis is CD133) shows that CD133+ cells make up 1.36% of all DTCs.

CD133 expression was analyzed in 22 BM samples. Half of these BM samples were DTC-positive. There was a single DTC-positive sample containing a CD133+-DTC subset, which accounted for 1.38% of all DTCs in this case (Figure 3).

We have demonstrated that both the primary tumor and DTCs in BM may have a heterogeneous composition and express various antigens. The significance of this finding for the disease course and prognosis deserves assessment in further studies.

## 4. Discussion

BM as a niche for micrometastasis plays a key role in hematogeneous dissemination. By creating a unique microenvironment for tumor cells BM maintains their proliferative potential for many years. Disease recurrence decades after treatment of the primary is described for many entities, and skin melanoma is not an exception. Forty percent of skin melanoma patients develop distant metastases at five or more years after curative surgery [27], therefore finding novel factors for disease prognosis and markers of early tumor cell dissemination for personalization of early systemic treatment is of much importance.

As demonstrated in our study, flow cytometry with a specific antibody HMB-45 in combination with CD45 is a useful technique to assess BM involvement in melanoma. DTCs were found in 57.4% of skin melanoma cases. The DTCs were present in 28.6% of stage I disease, which confirms the aggressiveness of skin melanoma even in localized disease. The findings of CD57 and CD133 expression are evidence of DTCs heterogeneity and the complex hierarchical relations between the primary and the DTCs. The prognostic significance of our results will be assessed in further studies.

Thus, we can talk about the complex relationship of hematopoiesis in general and the development of skin melanoma. Both myelo- and erythropoiesis are involved in and reacting to the tumor process occurring in the body. Of particular interest are changes in the bone marrow hematopoiesis arising in the presence of DTCs. Perhaps they are a reflection of the reorganization of the microenvironment of the DTCs, contributing to their long-term persistence in the bone marrow. The role of these changes in the prognosis of the course of the disease remains to be assessed.

The ‘seed and soil’ theory therefore is still valuable after 150 years and requires further development using up-to-date diagnostic approaches.

## Figures and Tables

**Table 1:** The distribution of patients by disease stage.

Stage	Frequency	Percent (%)
I	7	14.9
IIa	1	2.1
IIb	5	10.6
IIc	3	6.4
III	11	23.4
IV	20	42.6
Total:	47	100

**Table 2:** Monoclonal antibodies used in the study.

No.	MoAbs/Fluorochromes	Function	Manufacturer
1	Syto41	Nuclear dye	Thermo Fisher Scientific, Walthem, MA, USA
2	CD45	Leukocyte common antigen	Beckton Dickinson
3	HMB-45	Melanoma cell antigen gp100	Santa Cruz Biotechnology, Dallas, Tx, USA
4	CD56	Neuronal cell adhesion molecule (NCAM)	Beckton Dickinson
5	CD57	NK-cell molecule (HNK1)	Beckton Dickinson
6	CD133	Hematopoietic stem cell antigen	Beckton Dickinson

**Table 3:** Comparison of the average bone marrow according to myelogram.

Myelogram Parameters	DTCs	*n*	Mean Value	Err^std^mean	*p*
Cellularity	negative	19	67.0	6.51	NS*
	positive	20	67.3	7.87	
Blasts	negative	20	1.46	0.14	0.026
	positive	25	1.09	0.09	
Promyelocytes	negative	20	0.44	0.11	NS
	positive	25	0.37	0.08	
Neutrophilic myelocytes	negative	20	7.80	0.72	NS
	positive	25	8.95	0.54	
Neutrophilic metamyelocytes	negative	20	8.58	0.65	NS
	positive	25	7.83	0.53	
Band neutrophils	negative	20	16.50	0.91	NS
	positive	25	18.70	1.00	
Segmented neutrophils	negative	20	24.47	1.39	NS
	positive	25	27.266	1.71	
All granulocyte cells	negative	20	60.76	1.45	0.025
	positive	25	65.41	1.38	
Neutrophil maturation index	negative	20	0.43	0.034	NS
	positive	25	0.38	0.034	
Monocytes	negative	20	2.78	0.26	NS
	positive	25	3.30	0.24	
Lymphocytes	negative	20	12.85	0.79	NS
	positive	25	12.02	0.68	
Plasmocytes	negative	20	0.60	0.10	NS
	positive	25	0.77	0.15	
Basophilic normoblasts	negative	20	1.23	0.17	NS
	positive	25	0.97	0.13	
Polychromatophilic normoblasts	negative	20	11.16	0.91	NS
	positive	25	10.19	0.82	
Oxyphilic normoblasts	negative	20	9.08	0.88	0.006
	positive	25	6.25	0.52	
Sum of nucleated erythroid cells	negative	20	21.47	1.44	0.042
	positive	25	17.41	1.29	
Erythroid maturation index	negative	20	0.96	0.01	NS
	positive	25	0.96	0.01	
Leuco–erythroid ratio	negative	20	4.02	0.39	0.034
	positive	25	5.58	0.59	

* NS—not significant.

**Table 4:** Frequency of disseminated tumor cell (DTC) detection at various stages of melanoma.

Stage	Number of Patients	Frequency of DTCs + Cases
I	7	28.6%
II	9	55.6%
III	11	63.6%
IV	20	65.0%
